# Integrated community based child survival, reproductive health and water and sanitation program in Mkuranga district, Tanzania: a replicable model of good practices in community based health care

**Published:** 2012-12-26

**Authors:** Koronel Mashalla Kema, Joseph Komwihangiro, Saltiel Kimaro

**Affiliations:** 1 African Medical and Research Foundation (AMREF), Tanzania Country Office, Dar es Salaam, Tanzania

**Keywords:** Child survival, community based, integrated health care, reproductive health, water and sanitation, Tanzania

## Abstract

**Background:**

Over decades, evidence has accumulated to justify the concern that top-down approaches do not work and may result in lack of program ownership and sustainability. As a result, participatory approaches have increasingly become popular. An example of such an approach is hereby presented.

**Description:**

Working with AMREF, Mkuranga district significantly gained experience and improved its community participatory approaches in health development. AMREF's model of Community Based Health Care (CBHC) approaches was used to implement integrated Water and Sanitation, Child Survival and Reproductive health programs.

**Outcomes:**

The project established functioning village health and water committees. A 45% increase in utilization of services was reported. Adequate nutrition status among children rose from 67.9% to 81%. Attendance of antenatal clinics rose from 35% to 70.2%. A total of 117 shallow wells, 21 boreholes and 25 rain water harvesting systems were established.

**Lessons learnt:**

Based on this experience, we conclude that in order to achieve the Millennium Development Goals (MDGs) and the National Poverty Reduction Strategy (Mkukuta) targets, building partnerships with communities who are the target beneficiaries is a prerequisite and CBHC approach is a fundamental towards attaining those goals.

**Conclusion:**

The model demonstrates that community participation is key to community empowerment, as well as community ownership and sustainability of health interventions.

## Background

Mkuranga District is one of the six districts of Tanzania′s Coast Region. It is about 50 km South of Dar es Salaam, along the Kibiti - Lindi highway, connecting Tanzania to Mozambique. In 2002, its population was about 197,900 people [1]. Mkuranga population was by then served by 32 public and private health facilities, including one district hospital, two health centers and 29 dispensaries. On average, one health facility serves about 6,100 people. For a long time Mkuranga was characterized by marginalized communities, difficult-to-reach areas and limited access to health services. This deprived communities of their fundamental human rights. A situation analysis that was conducted in 1999 during the pilot inception of the Water and Sanitation project revealed a number of key health indicators. Infant Mortality Rate (IMR) was estimated at 120/1000 live births while the national average was 88/1000 live births [1]. Under-five mortality rate was estimated at 202/1000 live births against the national average which was 137/1000 live births [2]. Malaria was identified to be the leading cause of under-five deaths in the district and ITN use was about 5.1% [2]. The proportion of households with access to safe water from protected sources was estimated at about 25% for the entire district and 4-10% in the rural areas, while the national average for rural areas was 40% [3]. Most households used unsafe water from unprotected sources, with high risk of contamination, especially during rainy seasons. Wild animals such as baboons also contaminated the water as they drank from the same sources. Maternal Mortality Ratio (MMR) was high, though not well documented. Proxy indicators suggested a far higher rate than was then the national average of 529/100,000 live birth [3]. With regard to live birth, a reproductive health baseline survey in Mkuranga revealed that only 32.1% of deliveries occurred at the health facilities. The survey further revealed that maternal death was common in Mkuranga: 65% of respondents knew of a woman who had died of pregnancy and childbirth related complications and 78% of them knew of more than one case.

The health systems and infrastructure were poor. Primary school enrolment was low and the dropout rate of girls in some schools was as high as 53%. Traditional beliefs and practices were prevalent, gender imbalance was high. About 96% of children had undergone ovuletomy, a bad practice of cutting the child's uvula (epiglottis) when a child has prolonged fever and cough [2], thus leading to severe anaemia due to excessive bleeding and consequently death.

The partnership between AMREF and Mkuranga district council started in 1999 when AMREF responded to the alarming health and development situation in the district [4]. The aim was to support the district to develop community health interventions to meet the basic needs of the community. The target area for the intervention was 24 rural villages in four wards of Mkamba, Panzuo, Shungubweni and Mbezi. This area is geographically remote and hard-to-reach, with poor socio - economic condition. The target population for the project are was about 32,000 people, whereas 9000 were women of child bearing age, 6000 children below five years of age, 13,000 men in the community and ultimately the entire population (187,900) in Mkuranga District. The summary information for the three projects is presented in Table 1.

**Table 1:** Summary of project information and objectives

The Water, Hygiene and Sanitation Project	
**Budget**	US$ 755,853
**Dates**	January 2001 - December 2005
**Donor(s)**	AMRE in Italy, The Big Lottery Fund, Diego
**Partners**	Mkuranga District, Council, the communities and AMREF in Tanzania
**Goal**	To contribute to a reduction in the burden of disease in the community while at the same time reducing the work burden on women.
**Objectives**	To increase access to potable water to at least 70% of the households To establish community mechanisms and capacity to provide water and sanitation facilities To improve household knowledge and behavior in latrine use, ITN use and appropriate solid and liquid waste disposal.
**The Child Survival Project**	
**Dates**	July 2002 - June 2004
**Donor(s)**	Madrid City Hall, AMREF in Spain, AMREF in Tanzania
**Partners**	Mkuranga District Council, the communities and AMREF in Tanzania
**Goal**	To improve health of children under -five years of age by reducing their morbidity and mortality rates in targeted communities
**Objectives**	To strengthen community structures in at least 75% of the target villages to support child care practices at the household level To improve the health seeking behavior among at least 60% of parents and other caregivers of children under- five years old in targeted communities To strengthen linkages between community based institutions such as schools, health facilities, and others to enable households in at least 50% of the targeted communities to fulfill their responsibilities towards child health To facilitate the Health Care Providers to provide Integrated Management of Childhood Illness.
**The Reproductive Health Project**	
**Dates**	Start date: 01/07/2004 End: 15/09/2007
**Donor(s)**	The Health Foundation (UK); The Bush Hospital Foundation (UK), Direct Relief International (DRI) and AMREF in UK, AMREF in Netherlands
**Partners**	Mkuranga District Council, the communities and AMREF in Tanzania
**Goal**	To improve the health status of women in Mkuranga District within the context of women's rights and reducing maternal Morbidity and mortality
**Objectives**	To increase access to and utilization of health facilities for maternal health services (including family planning) for women of child - bearing age living in Mkuranga district -Tanzania. To increase the capacity of community structures to support and provide effective maternal health care To increase the involvement of men in maternal health services To advocate for an environment that is sensitive to the needs of women, with particular reference to maternal health at local and national levels.

In 2001 AMREF initiated a 5-year Water, Hygiene and Sanitation (Watsan) project in the four wards, following a one year pilot study that was done in Mbezi Ward. The project focused on developing and testing models and approaches which work effectively in rural and poor income urban areas to increase access to safe water and improve sanitation [4]. The approach used simple but evidence-based technology that is acceptable, affordable and sustainable to the local population. The project emphasis was on capacity building for district and community structures to own and manage water and sanitation facilities including operation, maintenance and financial sustainability.

In response to the community demand during the implementation of the Watsan project and following the building and strengthening of community structures and systems [5], in 2002 AMREF introduced a two-year child survival and reproductive health intervention in the same target area. The project focus was to pilot a community-Integrated Management of Childhood Illnesses (c-IMCI) in poor communities as a means of reducing under - five morbidity and mortality rates.

The child survival and reproductive health projects had two major components, namely the community and clinical components. The community component emphasized on strengthening the community structures and equipping them with skills to mobilize and sensitize the beneficiaries to actively participate in identifying and solving their reproductive and child health problems, including demand creation and increasing access and promoting utilization of services provided within the formal health systems. The clinical (health facility) component focused on strengthening the health systems and building the capacity of the Health Service Providers to provide comprehensive, accessible and improved quality reproductive health care [6]. By implementing the two components, the project increased the link between the community and the formal health system and minimized the gap between the two.

## Description

### Implementation approach

**Figure 1:**
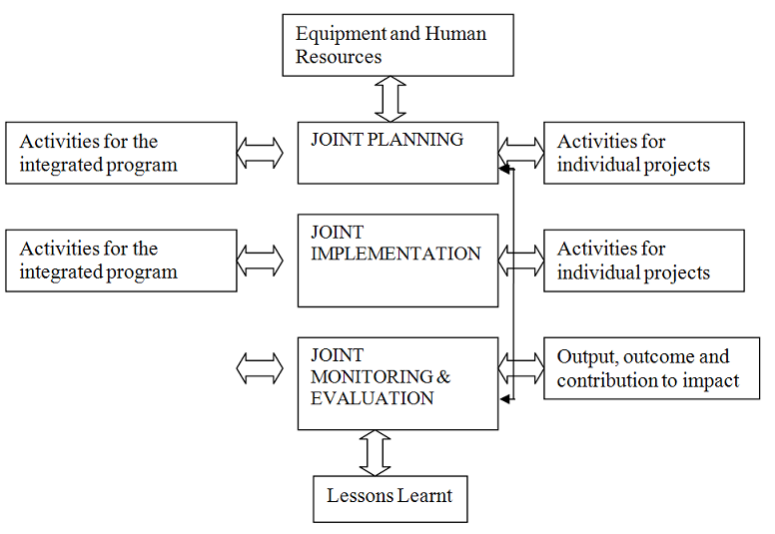
Mkuranga Program integration framework

Community Based Health Care (CBHC) was the fundamental implementation approach for this program. The three projects were integrated into one comprehensive program to enhance the synergistic effect on impact and costs. The integration framework is summarized in Figure 1.

### Enhancing community participation

AMREF′s approach used community forums, inter-village competition, community theatre groups, village health days and house-to-house visits as broad community mobilization approaches.

*Community forums:* Different forums were organized for purposes of community mobilization. Such forums included Village General Assembly, Village Council Meetings, Ward Development Committee (WDC) meetings, hamlet general meetings and public rallies. Messages conveyed during these forums depended on the audience and the stage of the project in its cycle management.

*Inter-village competitions:* An innovative approach used in Mkuranga for Community mobilization was through inter-village competitions in order to promote hygiene and sanitation as well as increase male involvement in reproductive and child health issues. Competitions for hygiene and sanitation promotion were conducted with support of the community leaders and Community Own Resource Person (CORPs) by using a standard check list for assessing hygiene and sanitation practices at the household level.

*Community theatre groups:* Cultural groups were used for community mobilization as well as for communicating different health messages. Most villages in the project area had cultural groups and the project only strengthened their capacity to effectively mobilize communities on behaviour change and deliver health messages through different methods such as songs, role plays and other types of drama. *Village Health Days:* Conducting Village Health Days was another community mobilization approach that the project used to reach more people with integrated health information massages and services on a single occasion. *House-to-house visits:* Household visits were carried out regularly by CORPs with support from the Steering Committee and the project staff. These household visits provided an opportunity for direct interaction between the CORPs, project team and household members. *Construction of water and sanitation facilities:* As a means of improving access to clean and safe water supplies, wells were constructed (includes boreholes, shallow wells and protecting of springs). Sanitation promotion was done at household level as to improve sanitary conditions and improve hygiene behaviour practices among community members.

### Empowerment of communities through capacity building

A training curriculum and different training packages were developed and used to train different committees, with the aim of strengthening health management systems for community and district leadership on prevention and control of communicable diseases. Contents of the training included, gender based intervention approaches, resource mobilization and life saving skills for health care providers. The different committees received training as a capacity building strategy to strengthen health system including:

*Council Health Management Team (CHMT):* The CHMT is the technical organ responsible for managing health programs in the district. CHMT supports and oversees health interventions in the district, including those at the peripheral health facilities.

*Project Steering Committee:* was established to support a smooth implementation and follow up of the project. This committee was multi-sectoral in composition, consisting of personnel from various sectors that include health, community development, planning, education, agriculture and water.

*Ward Development Committee (WDC):* a government structure that oversees all development programs at the ward level. It was multi-sectoral in nature, composed of village leaders and extension workers (agriculture, health, education, community development etc) at ward level with the ward counselor as the chair. In Mkuranga, one CORP from each program village was a co-opted member of the committee to ensure effective management, accountability and reporting of program activities at ward level.

*Village Council:* a government structure that oversees all development programs at village level. It was made up of village and hamlet leaders and key persons selected during the general village assembly. In Mkuranga, at least one CORP was a co-opted member of the village council to ensure effective management, accountability and reporting of program activities to village leaders.

*Village Water Committees:* a government structure formed by and accountable to the village council. It consisted of eight gender-balanced members. Its overall task was to oversee all water and sanitation related issues in the village. It supported the implementation of activities such as the identification of well construction sites, construction of the wells, mobilization and management of water funds and maintenance of water points.

**Iddi Lusambi**, 39, is a Ward Executive Officer (WEO) for Panzuo Ward in Mkuranga district. He is married with four children. Besides his employment, he supplements his income through subsistence farming. Talking about the quality of health services at Kibuyuni dispensary, Lusambi narrated this:

“Before the start of the reproductive health project the condition of our dispensary was very bad. The building was old with cracks and limited space to accommodate all the required services that are provided at the dispensary. There was not enough equipment to provide quality care for pregnant mothers. The situation has significantly changed after the project supported the construction of a new maternity home building”

Regarding community participation, Lusambi reported that, the community was fully involved in the project. He said, *“Community members contributed building materials like sand, water, manpower for brick making and even cash contributions to buy fuel and pay for the driver′s allowances”. He further said that, “By virtue of my position, I was very much involved in program activities especially in mobilizing communities as well as coordinating the village leaders”.*

Commenting on some of the achievements by the project, Lusambi said that many community members through CBHC approach had been sensitized and motivated to take action in improving their own health as also evidenced in the construction of wells and contributions into the community water fund. He further observed that *“the vaccination coverage of children has increased, people now access clean water and more women deliver at our dispensary”*.

*Village Health Committees:* also a government structure formed by and accountable to the village council. It was made up of at least nine members with gender balance. Its main role was to oversee all health related issues in the village. It also supported the CORPs in their work.

### The drive behind community owned resource persons’ motivation to work as volunteers

CORPs are the primary implementers of program activities in their respective villages [7, 8]. The CORPs may also collect and manage community based data and communicate the data to the higher levels to facilitate bottom-up data guided planning. The CORPs are the key entry point to the community.

**Selection of the CORPs:** The health and development program in Mkuranga was implemented through community volunteers, the majority of whom were men and women aged between 18-30 years, and a few above 40 year of age. Some were married and had family responsibilities. CORPs were selected by community members following set criteria agreed upon by the community through the WDC and the program team. The criteria include ability to read and write, living in the respective village, willingness to work as a volunteer and acceptability of an individual by the community. *Training the CORPs:* Training curriculum and materials were developed to facilitate the training process. The initial training package was broad enough to equip the CORPs with knowledge and skills to recognize health as a broad social development phenomenon. Training was conducted for 21.days. *Key tasks performed by CORPs:* The CORPs worked within the framework of the district health systems and priorities. Their work complemented efforts of the formal health systems and they received support from relevant technical experts within the community as well as from the project. The tasks performed by the CORPs in the Mkuranga program included community mobilization and sensitization on program activities, organizing the Village Health Days, house-to-house visits, collecting information and writing reports. *What motivates CORPs to work as volunteers’* CORPs are purely volunteers. However, sustaining voluntarism among CORPs without any incentives has been a global challenge. It requires innovative and creative approaches. In the Mkuranga program, it was learnt that the volunteerism spirit among CORPs was maintained and sustained even without financial incentives through the following: *Community involvement and participation at project inception:* During the introduction of the project in the community, open and transparent discussions were conducted between community members, the Programme team and the district authority. *Capacity building:* Training packages were developed based on programme objectives and knowledge needs of the volunteers. Health was addressed as broad community development issue and this motivated the CORPs to deal with a development agenda rather than focusing on diseases alone in a vertical approach.

**Supportive supervision:** Close and constant supportive supervision is a major motivating factor for CORPs. During the supervision, performance gaps by CORPs related to their tasks are addressed and this increases their confidence. Supervision also provides moral support as it showed that what CORPs were doing was supported by authorities and experts from higher levels. This increased their recognition, trust, confidence and morale. *Participation in project formulation and implementation:* CORPs, being part of the community, were not only involved at the implementation level, but also at the level of the project formulation. CORPs mobilizes community members and participates in identifying issues to be addressed. CORPs also participated in regular monitoring and evaluation through project reviews, data collection and reporting.

**Flexible time table:** CORPs were empowered to make decisions according to their situation and circumstances. Decisions are made within the broad framework of the program objectives. This allows flexibility in the timing of performing certain tasks without interfering with their livelihood activities. In this way, CORPs feels trusted and recognized by the Programme, communities and professionals.

**Recognition in the community:** Empowerment enhanced recognition of CORPs within their communities. Community members, the Programme team and higher administrative levels in the district recognize the CORPs′ contribution in community health development interventions.

**Other incentives:** Having worked for a significant period of time, the CORPs were awarded by the program certificates of appreciation and participation. This was a symbol of recognition for the services they render to the community and the program was an important motivation factor. Other incentives include visual identification materials such as T-shirts, bags, IEC matials, document folders and bicycles to facilitate transport during their work.

### Community participation in developing and disseminating information, education and communication (IEC) materials

Information Education and Communication materials are designed to provoke discussions and dialogue on different issues among community members and sometimes with the professionals. IEC materials may be presented in different ways that may include print and electronic forms.

**Figure 2:**
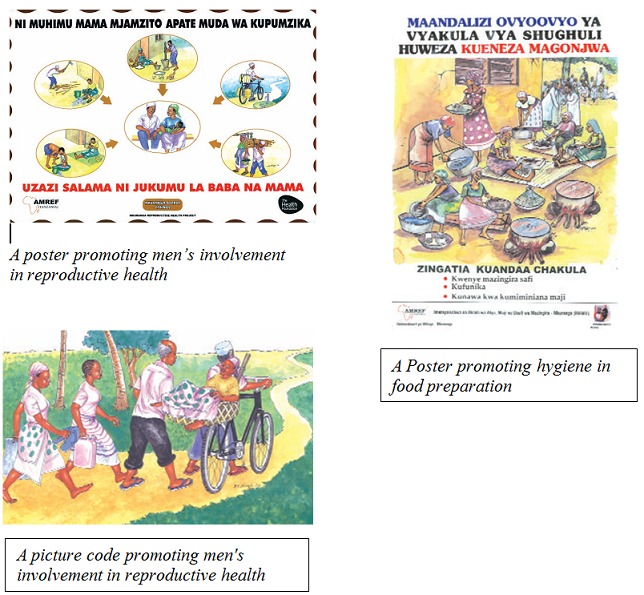
Some training materials used for IEC

*The process of developing IEC materials in Mkuranga Program:* A participatory workshop was organized to analyze and identify the issues to be addressed through IEC materials. Such a workshop comprises of different stakeholders, including the beneficiaries and program staff. During this workshop, the form of presentation (e.g. posters, leaflets etc) is agreed upon. Following identification of critical information and education gaps, areas which require IEC materials are prioritized based on the magnitude of the problem and the ability of the program to address the problem through IEC materials (Figure 2). *Dissemination* of the IEC materials is carried out in several ways that include the use of forums such as Village Health Days, football tournaments, workshops and seminars, mounting of posters and wall murals and in public areas such as schools, and health facilities.

**Shabani Digubike**, 42yrs old, is a CORP for Kizomla Village. After three years of involvement in program activities, he was selected a Village Executive Officer (VEO) for the same village. He is married with one child. Before his involvement with AMREF activities, he worked as a village health worker with support from the district council. *“I was selected to become a VEO because I was trusted by the community to undertake different activities, especially after attending different training opportunities organized by the project”*, says Mr. Digubike. Mr. Digubike further reports that he was involved in mobilizing communities for different activities, including construction of a new maternity waiting home at Kibuyuni dispensary. He compiled data from CORPS and writes reports for his village. *“The construction of the maternity home at Kibuyuni has motivated pregnant women in this village to deliver at the clinic as they now have a place to stay while waiting and after delivery. Before construction of the facility and due to the long distance from the dispensary, women used to deliver at home. The six water wells, which have been constructed in our village, have increased our access to clean and safe water”*. Mr. Digubike narrated. According to Mr. Digubike, the Village Health Days have helped to increase the vaccination coverage for young children. *“People have agreed to contribute funds for maintenance of pumps and wells and each woman who comes for Village Heath Day contributes Tsh 50/ (about US $ 0.04) to support the event” At a personal level Mr. Digubike said; “through AMREF I have acquired knowledge and skills for community mobilization and how to facilitate community development activities. That is probably why they selected me to be their leader.”*

### Village health days: an effective approach to reaching communities

The revamping or introduction of the Village Health Days by the program in Mkuranga was done in three phases: Phase I: Conducting Village Health Days at Ward Level: The aim was to pilot how the occasion should be organized in different villages and served as an opportunity for capacity building. Phase II: Conducting Village Health Days at Village Level: These were initially conducted on a monthly basis but later on they were scheduled to take place on quarterly basis. Phase III: Conducting Village Health Days at hamlet level: In some villages, Village Health Days were also conducted at the hamlet level in order to reach as many people as possible.

*Mobilizing communities for a Village Health Day:* some preparatory activities are made and these included holding meetings and mobilizing resources. The CORPs and the community leaders are the key people involved in the preparatory meetings.

*Activities carried out during the VHD:* Due to mass turn up during the event, sometimes CORPs from other villages in the neighborhood volunteers to support their colleagues in facilitating the event. Key events include a key note address on health matters normally given by a village leader or any other designated person invited to do so.

**Rebecca Sanga** is an employee of Mkuranga District Council and has been working as a public health nurse at Kibuyuni dispensary since April 2002. She is the first born in a family of four. Besides her employment, she sustains her living by running a food kiosk, which is attended by her sister. She also gets a commission by selling Hati Punguzo ITN vouchers through the National Voucher Scheme Program. Rebecca has been involved with AMREF program interventions since 2002, initially as a CORP and currently as a district Trainer of Trainers (TOT), having received some training through the AMREF program. Regarding her involvement in Village Health Days she has the following to say: *“A Village Health Day (VHD) is a special day, agreed upon with community members. On that day, children are weighed and vaccinated. Health education and demonstrations are provided to the community on nutrition”*. Rebecca gives an account of her involvement during the VHD: *“My key responsibilities during the VHD include weighing of children and attending pregnant mothers, immunization, providing community health education on maternal and child health and sanitation, and health education through demonstration e.g. preparation of ORS, treatment of ITNs, and preparation of nutritious meals for kids. I also treat (or refer) sick children and pregnant mothers and provide Family Planning methods.”* Commenting on the success gained from conducting VHDs as a community mobilization approach, Rebecca narrates, *“Attendance at my clinic has tremendously increased. On average attendance at MCH clinics is 50-60 children and 20-30 pregnant women per day. Previously (2002 - 2003) only 10 - 15 pregnant mothers attended the clinic. During the rainy season, attendance tends to drop. During the VHD, 100 - 120 children are reached with MCH services compared to only 50-60 children before the VHDs were strengthened”*. Rebecca also gives an account of other program achievements that include hand washing habits and more women choosing to deliver at the dispensary. At a personal level, Rebecca says she has benefited by understanding better her working environment and has gained more knowledge and skills on handling complicated deliveries.

*Community health education:* Commonly used methods were health talks and demonstrations. Health education covers issues related to water, hygiene and sanitation including water point protection such as pump maintenance and repair.

### Sustainability of Community Based Interventions

The following initiatives were put in place to enhance sustainability of the interventions:

*Formation of multi-sectoral steering committees at district level:* After a sensitization meeting with district level stakeholders on the program, a multisectoral steering committee was formed. The steering committee is responsible for supporting and management of program implementation processes as well as provision of technical support to the CORPs and the communities.

*Private Public Community Partnership:* Public private partnership was demonstrated in some villages where private agents (individuals) are commissioned to manage the water wells which have been constructed with support from the program. The agent is responsible for security and minor repairs of the well and pump.

**Figure 3:**
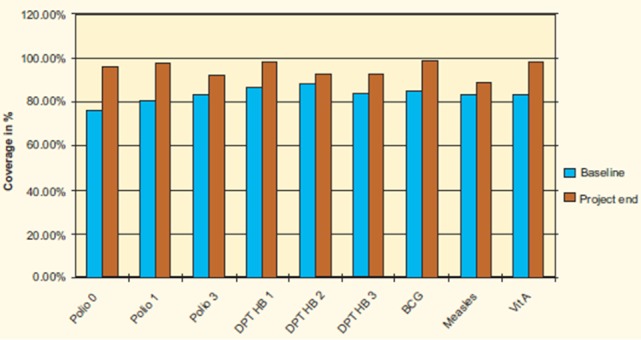
Change in vaccination coverage following project implementation

*Use of local resources and technology:* Locally available materials and appropriate technology is used to promote and achieve sanitation and hygiene objectives. Timber boxes and ′madema′ are used to construct pit latrines (Figure 3) in areas with sandy soil to avoid collapsing of the pit. Such materials replace the use of cement and blocks which are expensive and unaffordable by most community members.

*Capacity Building of Community Owned Resource Persons (CORPs):* Different groups of CORPs are trained by the program to carry out different program tasks. They were responsible to the village councils in their respective villages. An average of ten Community Health Workers (CORPs) one supervisor and a TOT have been trained in each village to keep alive the project activities beyond the funded project period.

*Capacity building and use of existing local and district structures:* Capacity building of different community structures to later manage the project was implemented through workshops, project reviews, and study tours where beneficiaries were also fully involved.

## Challenges

Poor commitment of some community leaders associated with lack of transparency and lack of adequate communication skills hampered implementation. Similarly, prolonged dry season make some shallow wells to dry up in the region. Some communities are difficult to reach especially during rainy seasons. This interferes with sustainable monitoring and supportive supervision by the district team.

Some villages are large and households are scattered. Some parts of the villages are not easily accessible especially during rainy seasons. To overcome this limitation, the program provided each village with a bicycle to facilitate the CORPs with transport to reach remote communities in the project area. Change of political and administrative leadership in the project areas necessitates repeated sensitization and capacity building of the new leadership at community and district levels. Low literacy level (women 51% and men 61%) compounds the problem of entrenched traditional cultural practices and beliefs, making behavioral change even more difficult. Likewise, limited basic education further complicates capacity building processes.

## Outcomes

This section summarizes the key outcomes, contributing to impacts and lessons learnt during the implementation of the Mkuranga program.

### Community structures and health systems

Community structures and health systems were strengthened and linked to each other [9]. The project notes that target villages had functioning village health and water committees which were linked and accountable to the village government council. The committees held monthly meetings and recommendations from these meetings feed into ward and district level meetings. In this way, community voices are heard in district planning processes. A Community Based Health Management Information System (CBHMIS) was well established and functioning at 100% of the target villages. Reports are then channeled through the village leadership to the nearby health facility, AMREF and the District Medical Officer at district level. This data provides the information required for evidence-based district planning.

A total of 96% of the community owned resource persons were retained over the period of five years. Additional CORPs were recruited on request. The project started with 264 CORPs and TOTs in 2002 and this number increased to 312. The dropout rate of 4% was reasonable and insignificant. The district had adopted and scaled up the intervention packages to cover the entire district. The Life Saving Skills and clinical c-IMCI packages were implemented at 100% of the health facilities in the district, including those owned by the private sector.

**Theresia Mumbi** is a 39 year old Nurse Attendant at Msorwa dispensary. She has been working at the dispensary since 1989 when she finished her primary school education. Theresia has never received any formal nursing training - she was employed at the dispensary as soon as she completed her primary school education and has simply been trained ′on the job′. Msorwa dispensary serves up to about 8,116 people, yet it only employs three members of staff - one Assistant Clinical Officer and two Nurse Attendants. *“Due to this serious understaffing, I have been doing various activities including conducting deliveries and dispensing etc, even though I am not trained to do so. All this is in order to save lives”*, says Theresia. Indeed, Theresia is in charge of all reproductive and child health activities at Msorwa dispensary. She does all the duties that a Public Health Nurse would do if one was available. Yet for many years, the District Council has failed to employ one for this dispensary.

Theresia was nominated by the project to attend the 21-day training in Life Saving Skills in obstetric and newborn emergencies. Other people involved in this training included Clinical Officers, Nurse Midwives and Public Health Nurses. *“Initially I felt out of my depth. Everyone was far more qualified than I, and I thought that I could not make it. But I said to myself, I must try”*, said Theresia. *“The training has changed my life. It has equipped me with new skills and has significantly built myself confidence. This was the longest and most intensive training I have ever received since I finished my primary education. Now I know a lot more and most importantly, I feel much more confident”*, says Theresia.

### Child survival

**Figure 4:**
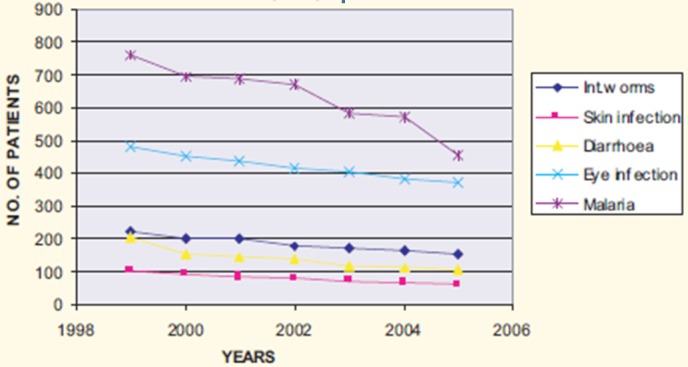
Water-related diseases among children under five years at Msorwa Dispensary

The program resulted in an increase in utilization of health services that are provided at the health facilities by 45%. A decrease of about 90% in the proportion of children arriving at the health facilities with severe disease conditions (e.g. febrile convulsion, severe dehydration from diarrhea and severe pneumonia) has also been recorded. Vaccination coverage increased from an average of about 76% to over 98% (Figure 3). The project improved the children′s nutritional status. The proportion of children with good nutrition status (based on weight for age) increased from 67.9% to 81% while the mild nutrition decreased from 29% to 16%. The proportion of children with severe malnutrition did not change as it remained at 3.1% and 3.0% at baseline and project end respectively. Remarkable reduction in morbidity was recorded among under-fives as shown in Figure 4. The demographic health survey in 2004 documented Under-five Mortality Rate of 174/1000 live births compared to 220/1000 live births in 2000 when the project was introduced.

### Maternal health

**Figure 5:**
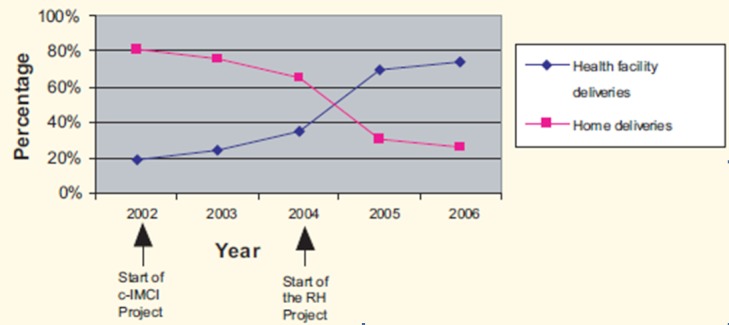
Trend of health facility deliveries at Mkamba Health Centre

Utilization of health facilities for maternal health care improved (Figure 5). Nearly 100% of women visit antenatal clinics at least once in the course of pregnancy and early booking (starting ANC before gestation age of 20 wks) increased from 35% at baseline in 200 to 70.2% in 2007. The project supported construction of maternity waiting homes at nine rural health facilities. The proportion of women delivering their babies at the health facilities increased from 26.3% at baseline in 2004 to 70.4% in 2006 in the intervention area [10]. The national average for this data is 47% [11] and Mkukuta target by 2010 is 80% [12].

Quality of maternity services at the health facilities had improved. A total of 97% of women were satisfied with the quality of maternity services compared to 47% at baseline in 2004.The proportion of households owning at least one bed net has increased from less than 5% in 2002 to 85.4% 2007. Men are increasingly involved in reproductive health matters. A total of 94% women now report adequate support from their male spouses during pregnancy compared to 47% at baseline in 2004. Men are increasingly accompanying spouses at ANC visits.

### Water and Sanitation

The project facilitated construction of 117 shallow wells, 21 bore holes, 25 rain water harvesting systems and protection of 2 natural springs. This led to an increase of the proportion of households with access to safe water from less than 25% in 2000 to 85.1% in 2006 when the project ended. The national average for this indicator is 53% and Mkukuta target for 2010 is 65% [12]. 100% of the target villages had established water funds and opened bank accounts.

All the project villages had at least three trained water artisans to support operation and maintenance of their water points. Basic sanitation improved. The proportion of households using pit latrines increased from 33% at baseline to 81% at project end; 62% and 91% households had ash pits and dish drying racks respectively.

## Lessons learnt

Community problems are complex and intertwined in a complex manner such that when people are empowered to solve one problem, their awareness increases and thus the demand to solve other problems increases as well. Community members have the potential to solve their health problems, but lack awareness of their potentials. Community health interventions should aim at creating an enabling environment for the communities to build competencies and realize their potential to bring about change. The decentralization policy renders power and responsibility to the community to plan and manage their own health development programs. However, their capacity is in most cases inadequate. Capacity strengthening for the community structures is therefore a prerequisite for programs which promote participation of the beneficiaries.

## Conclusion

Partnership in Community Based Health Care (CBHC) is an effective approach for implementing community health development, and may be scaled up and applied elsewhere to enhance community participation in other sectors as well.
